# Quantitative Analysis of Salivary TNF-*α* in Oral Lichen Planus Patients

**DOI:** 10.1155/2015/283465

**Published:** 2015-03-15

**Authors:** T. Malarkodi, S. Sathasivasubramanian

**Affiliations:** Department of Oral Medicine & Radiology, Faculty of Dental Sciences, Sri Ramachandra University, Porur, Chennai 600116, India

## Abstract

*Objective*. The aim of this study was to quantitatively evaluate the salivary tumor necrosis factor-alpha (TNF-*α*) level in oral lichen planus patients and to compare the levels of TNF-*α* between saliva and serum of OLP and controls. *Methods*. Serum and whole saliva from 30 patients with active lesions of oral lichen planus (OLP) and 30 healthy persons were investigated for the presence of TNF-*α* by enzyme immunoassay. Student's independent *t*-test and two-sample binomial proportion test were used to calculate significance of the mean values of TNF-alpha in serum and saliva and to determine the proportions of the detected and nondetected samples in both groups. *Results*. Proportion of detection and the mean of detectability between saliva and serum of Group B show an almost equal value, which suggests that saliva can be a good alternate to serum to analyze TNF-*α* in oral lichen planus patients.

## 1. Introduction

Lichen planus (LP) is a chronic inflammatory mucocutaneous disease. It was first described by the British physician Erasmus Wilson in 1869. The clinical appearances of these lesions resembled lichens a primitive organism of symbiotic algae and fungi growing on rocks, hence designated as lichen planus [1].

This disease primarily affects the skin and mucosal surfaces including the oral cavity. It can also involve other sites like the scalp and the nails [2]. It is estimated to affect 1% to 2.0% of the general population. The prevalence of oral lichen planus (OLP) in India is around 2.6%. Around 40% of lesions occur on both oral and cutaneous surfaces, 35% occurs on cutaneous surfaces alone, and 25% occur on oral mucosa alone [1]. Oral mucosal involvement can occur concurrently with cutaneous disease or it can be the only manifestation [3]. WHO has grouped this condition under the “potentially malignant disorders” of the oral mucosa [4].

Out of the six clinical forms of OLP the reticular, papular, and plaque type lesions are usually asymptomatic [5] whereas the erosive, atrophic, and bullous type lesions usually present with burning sensation and rarely with pain [3]. Malignant transformation or development of malignancy in the presence of OLP is more likely to occur in atrophic and erosive forms [1]. Majority of studies have reported the rate of malignant transformation of OLP to be between 0.5 and 2% [6].

The exact cause of LP remains unclear; however several predisposing factors like stress, chronic liver disease, and genetics have been implicated in the pathogenesis [3]. Current evidences suggest that LP is a T-cell mediated process. Adhesion molecules, cytokines, chemokine like intracellular adhesion molecules 1 (ICAM-1), vascular adhesion molecule (VCAM-1), interferon gamma (INF-*γ*) and tumor necrosis factor-alpha (TNF-*α*) serve to stimulate T-cells, initiate their adhesion to blood vessels, and direct their migration from the blood vessels to the tissues [1].

Tumor necrosis factor-alpha (TNF-*α*) has been implicated in the pathogenesis of many precancerous, cancerous, autoimmune, and inflammatory diseases including oral submucous fibrosis, oral leukoplakia, oral squamous cell carcinoma, systemic lupus erythematosus, rheumatoid arthritis, psoriasis, and OLP. In addition to being cytotoxic for certain tumor cells, it also acts as an essential mediator in inflammatory and immunologic reactions during host defense by increasing the major histocompatibility complex (MHC) class I antigen and intercellular adhesion molecule (ICAM) expression on many cells [1, 7].

Saliva as test specimen has several benefits over blood and is increasingly being used in the diagnosis of diseases. It offers distinctive advantages over serum because it can be collected noninvasively. Salivary analysis offers a reliable correlation of various parameters that are routinely evaluated in blood [8]. Early diagnosis and proper treatment are imperative as the oral lesion has the propensity for malignant development [9]. OLP is one of the most frequently encountered mucosal pathology by the dental practitioners. High concentration of TNF-*α* has a role in the progression of the pathological events in OLP [4] and only few studies are available in the English literature that has evaluated TNF-*α* in saliva of OLP patients. Hence this study was undertaken to evaluate the salivary and serum TNF-*α* levels in OLP patients and to probe whether saliva can be used as an alternative to serum in evaluating TNF-*α* in OLP patients.

The aim of this study was to quantitatively evaluate the salivary TNF-*α* level in oral lichen planus patients and to compare the levels of TNF-*α* between saliva and serum of OLP and controls.

## 2. Materials and Methods 

### 2.1. Subjects

The study group comprised sixty individuals, of which thirty were healthy volunteers and thirty were clinically and histopathologically diagnosed as OLP patients'. Women on oral contraceptives, pregnant women, smokers, alcoholics, and patients with liver disease, renal disease, diabetes mellitus, psoriasis, sjogren syndrome, rheumatoid arthritis, systemic lupus erythematosus, dental diseases, oral mucosal diseases, other infectious diseases, and history of trauma in the last six months, and patients under medications like dexamethasone were not included in the study. The patients' age group for the study ranged from 18 to 75 years with a mean age of 43.5. Out of the thirty oral lichen planus patients 16 were female and 14 were male. The clinical patterns of oral lichen planus were 22 with reticular type, 2 with erosive type, 4 with atrophic, and 2 with papular type. All the patients included in the study were those who had not undergone any form of therapy for their presenting illness. Their details were recorded in a specific case sheet pro forma, which was prepared earlier. Samples from thirty controls who participated in the study were drawn from healthy volunteers who were free of oral and medical illness. The age group for healthy volunteers ranged from 23 to 61 years with the mean age of 42. The patients and healthy controls were given explanation about the study and a written consent was taken. Procedures followed were in accordance with the ethical standards.

### 2.2. Collection of Samples

Blood samples were collected between 8:00 a.m. and 4:00 p.m. from the subjects. The blood samples were kept undisturbed for 30 min following which the serum was separated from the blood cells by centrifugation. The separated serum was stored in disposable plastic vials at −80°C until the time of analysis. 2 mL of whole unstimulated saliva were collected simultaneously by drooling method in sterile disposable plastic containers with lid. The salivary samples were transferred to sterile centrifuge tubes. After centrifugation the separated clear salivary fluid was stored in disposable storage vials at −80°C until the time of analysis. Care was taken not to collect sputum.

### 2.3. Cytokine Assay

All reagents and samples were brought to room temperature (18–25°C) before use. The samples were subjected to analysis and TNF-*α* concentration was determined using enzyme-linked immunosorbent assay kits (Ray Bio Human TNF-alpha Enzyme Immunoassay) and the results were interpreted by using spectrophotometry at 450 nm. On a linear graph, the optical density values were plotted for each of the calibrators (*y*-axis) against the corresponding standard concentration of TNF-*α* (*x*-axis), and a calibration curve was drawn. The TNF-*α* concentration of salivary and serum samples were calculated using the optical density and the concentration of the standard. The results were expressed as pg/mL.

### 2.4. Statistical Analysis

Statistical Package for Social Science (SPSS) software was used to analyze the data. Mean and standard deviation were estimated in each study group. The significance of the mean values of TNF-alpha in serum and saliva between the control and the patients were done using Student's independent *t*-test. The proportions of the detected and nondetected samples in both groups were determined using two-sample binomial proportion test.

## 3. Results

Students' independent *t*-test was used to compare the mean values of Group A (controls) and Group B (OLP) in Tables 1 and 2. As shown in Table 1, the mean value of salivary TNF-*α* in OLP patients was higher than the control group and it was statistically significant with a *P* value of 0.039.

As shown in Table 2 the mean serum TNF-*α* levels in OLP patients was higher than the control group and it was statistically very highly significant with a *P* value of 0.000697.

Two-sample binomial proportion tests were done to compare the proportion of detectability and nondetectability of TNF-*α* in salivary and serum samples.

**Figure 1:**
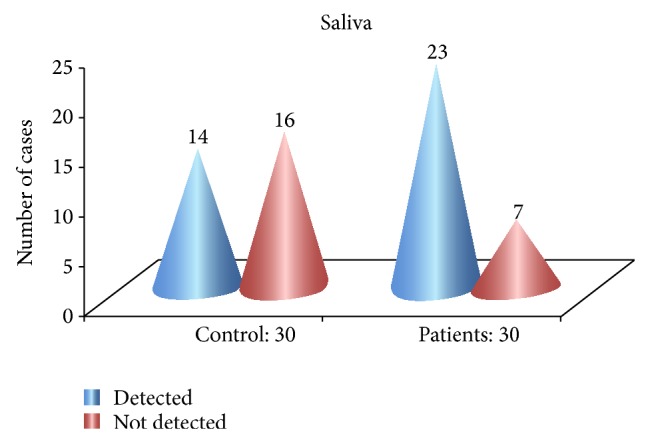
Proportion of detectability in saliva of Group A and Group B.

The proportion of detectability of TNF-*α* in saliva of OLP patients as shown in Table 3 and Figure 1 was high when compared to controls and was statistically significant with a *P* value of 0.02.

**Figure 2:**
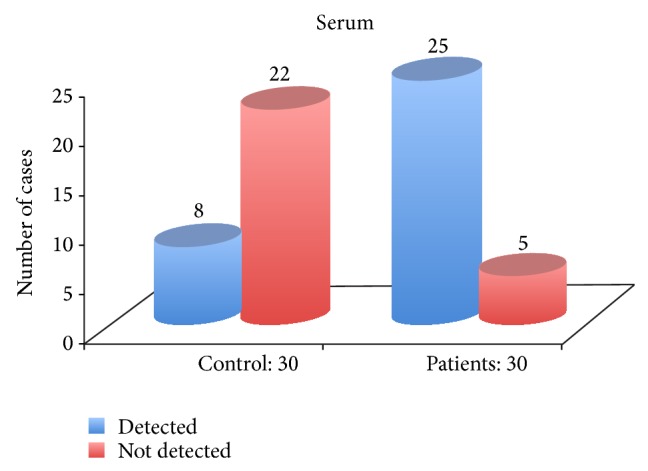
Proportion of detectability in serum of Group A and Group B.

The proportion of detectability of TNF-*α* in serum of OLP patients as shown in Table 4 and Figure 2 was high when compared to controls and it was statistically very highly significant with a *P* value of 0.001.

As shown in Table 5 the proportion of detectability of TNF-*α* in serum of OLP patients was high when compared with saliva from the same group but it was statistically not significant.

Students' independent *t*-test was used to compare the mean of detectability of TNF-*α* in saliva and serum of OLP patients. As shown in Table 6 the mean of detectability of TNF-*α* in saliva and serum of OLP patients is same.

Since there is no significant difference between the proportion of detection (Table 5) and the mean of detectability (Table 6) between saliva and serum of Group B, saliva can be used instead of serum to analyze TNF-*α* in oral lichen planus patients.

## 4. Discussion

Oral lichen planus (OLP) is a chronic inflammatory disorder. Although the exact etiology of OLP remains unclear, immunological aberration plays a demanding role among the various etiological factors [7]. Though controversies exist around the true malignant potential of OLP, WHO has grouped this lesion under the potentially malignant disorders of the oral mucosa [4]. Previous studies indicate a risk of 0.5–2% for malignant transformation [6]. Accordingly, there is a great need for objective markers to evaluate the prognosis of this condition. Since chronic inflammation has been suggested to be a cofactor for development of oral squamous cell carcinoma (OSCC) in OLP [9], the levels of proinflammatory marker TNF-*α* in OLP patients were evaluated in this study.

TNF-*α* is a potentially important and regulatory cytokine in the initiation and progression of OLP, Sugermann et al. [7]. According to Yamamoto et al. [10] and Sklavounou-Andrikopoulou et al. [11] high serum levels of TNF-*α* were detected in all patients with OLP in comparison with healthy controls. Simultaneously with the expression of other proinflammatory cytokines, OLP lesions have also been shown to contain cells with mRNA for TNF-*α* [12] and only few studies have measured the salivary levels of TNF-*α* in OLP patients [13–15]. As there are no studies available which compare the levels of TNF-*α* in saliva and serum in Indian population, the present study was carried out to evaluate and compare the levels of TNF-*α* in saliva and serum of oral lichen planus patients. The levels of TNF-*α* can be quantified using ELISA which is more sensitive, reliable, simple, and widely used and very little sample is required for estimation [16]. In the present study Ray Bio Human TNF-*α* ELISA kit was used to quantify the levels of TNF-*α*.

Body fluids that can be used to estimate the TNF-*α* levels are serum, saliva, and oral tissue transudates. The use of saliva as an adjunctive diagnostic tool has various advantages and supplements the current methodologies. Compared to serum, saliva has significant diagnostic and logistical advantage as a diagnostic fluid as it is less infective, noninvasive, relatively simple collected safely, collected repeatedly without discomfort to the patient, cost effective, easy and safe in disposal; does not require specialized training or special equipment; and lends itself readily for mass screening [17]. These advantages justify that saliva can be used as an easily accessible fluid for research purpose.

TNF-*α* from a variety of “resident” and “migratory” cells contributes to the total aggregation of bioactive TNF-*α* in OLP. Within the lesion T lymphocytes, mast cells and keratinocytes are considered to be the plausible cellular source of TNF-*α* [7]. The exact mechanism by which TNF-*α* enters saliva is by diffusion, active transport, and leakage through tissue transudates [18].

Unstimulated saliva better represents the physiological state when compared to a stimulated state [8]. Hence in the present study unstimulated saliva was used to determine the levels of TNF-*α* in saliva.

TNF-*α* value in Group B (OLP patients) ranged from 0 to 266.67 pg/mL in saliva and 0 to 246 pg/mL in serum. In Group A (controls) it ranged from 0 to 253.33 pg/mL in saliva and 0 to 236 pg/mL in serum.

Results suggested that the mean salivary levels of TNF-*α* was higher in Group B (OLP patients) when compared to Group A (controls) and it was statistically significant with a *P* value of 0.039. Similarly in serum, the mean value of TNF-*α* was higher in Group B (OLP patients) when compared to Group A (controls) and it was statistically very highly significant with a *P* value of 0.000697. These results were consistent with the previous studies done in saliva [13–15] and serum [10, 11, 19] and signifies that TNF-*α* plays an important role in the pathogenesis of OLP. The proportion of detectability of TNF-*α* in saliva of Group B (OLP patients) was significantly high when compared to Group A (controls) with a *P* value of 0.02. Similarly the proportion of detectability of TNF-*α* in serum of Group B (OLP patients) was very highly significant when compared to Group A (controls) with a *P* value of 0.001. The proportion of nondetectable salivary and serum samples for TNF-*α* was less in Group B (OLP patients) when compared to Group A (controls). Comparing the proportion of detectability between serum and saliva of Group B, (OLP patients) the detectability was marginally high in serum and the mean detectability level of TNF-*α* in saliva and serum of Group B (OLP patients) was same. This signifies that saliva can be a good alternate for serum to analyze TNF-*α* in oral lichen planus patients.

23.3% in saliva and 16.7% in serum were not detected for TNF-*α* in Group B (OLP patients). Diets rich in curcumin and yogurt has been shown to inhibit the activity of TNF-*α* levels in saliva and serum [20, 21] which can be considered as a reason for nondetectability of TNF-*α* in saliva and serum of Group B. Gavala et al. have reported that alcohol can inhibit the levels of TNF-*α* in blood [22]. Although alcoholic patients were excluded from the study, a possible explanation would be that some patients would have voluntarily abstained from revealing the habit. In spite of strictly following the required storage protocols low levels of TNF-*α* can occur during storage as they were estimated after a specific period of time. This can also be considered as a reason for nondetectability of TNF-*α* in saliva and serum of OLP patients.

Few salivary and serum samples in Group A (controls) showed elevated levels of TNF-*α*. Though the exclusion criteria were strictly followed, it can be suggested that these patients would have had a local trauma, aphthous ulcer, upper respiratory tract infection, or any other subclinical infection in the recent past for having high levels of TNF-*α* in saliva and serum.

Although patients with 5 different clinical manifestations of OLP were enrolled in this study, no subgroups were formed to categorize the clinical types to evaluate and compare the significant difference of TNF-*α* concentration between them.

This study proves the fact that the expressions of TNF-*α* in both the test specimens were almost equal. However larger sample size may be required to prove this in a conclusive manner. The study also points to a fact that saliva can be used as a test specimen in the evaluation of TNF-*α*.

## Figures and Tables

**Table 1:** Comparison of mean values of TNF-*α* in saliva of Group A and Group B.

Group	Number of cases	Mean	*t*-value	*P* value
A (controls)	30	30.55	2.11	0.039^*^
B (OLP patients)	30	63.22		

^*^Significant at *P* < 0.05; ^**^highly significant at *P* < 0.01; ^***^very highly significant at *P* < 0.001.

TNF: tumor necrosis factor.

OLP: oral lichen planus.

**Table 2:** Comparison of mean values of TNF-*α* in serum of Group A and Group B.

Group	Number of cases	Mean	*t*-value	*P* value
A (controls)	30	16.38	3.6	0.000697^***^
B (OLP patients)	30	68.44		

^*^Significant at *P* < 0.05; ^**^highly significant at *P* < 0.01; ^***^very highly significant at *P* < 0.001.

TNF: tumor necrosis factor.

OLP: oral lichen planus.

**Table 3:** Difference in proportion between detected and nondetected TNF-*α* values in saliva of Group A and Group B.

Group	Detected	Nondetected	Two-sample binomial proportion test	*P* value
A (controls)	14 (46.7%)	16 (53.3%)	*Z* = 2.38	0.02^*^
B (OLP patients)	23 (76.7%)	7 (23.3%)		

^*^Significant at *P* < 0.05; ^**^highly significant at *P* < 0.01; ^***^very highly significant at *P* < 0.001.

TNF: tumor necrosis factor.

OLP: oral lichen planus.

**Table 4:** Difference in proportion between detected and nondetected TNF-*α* values in serum of Group A and Group B.

Group	Detected	Nondetected	Two-sample binomial proportion test	*P * value
A (controls)	8 (26.7%)	22 (73.3%)	*Z* = 3.37	0.001^***^
B (OLP patients)	25 (83.3%)	5 (16.7%)		

^*^Significant at *P* < 0.05; ^**^highly significant at *P* < 0.01; ^***^very highly significant at *P* < 0.001.

TNF: tumor necrosis factor.

OLP: oral lichen planus.

**Table 5:** Difference in proportion between detected and nondetected TNF-*α* values in saliva and serum of Group B.

Group B (OLP patients)	Detected	Nondetected	Two-sample binomial proportion test	*P* value
Saliva	23 (76.7%)	7 (23.3%)	*Z* = 0.71	0.52
Serum	25 (83.3%)	5 (16.7%)		

^*^Significant at *P* < 0.05; ^**^highly significant at *P* < 0.01; ^***^very highly significant at *P* < 0.001.

TNF: tumor necrosis factor.

OLP: oral lichen planus.

**Table 6:** Comparison of mean values of TNF-*α* in saliva and serum of Group B.

Group B (OLP patients)	Mean of detected values	Standard deviation	*t*-test
Saliva	82.46	63.99	*t* = 0.02
Serum	82.13	61.86	

TNF: tumor necrosis factor.

OLP: oral lichen planus.
